# 

**Published:** 1918-03-09

**Authors:** 


### CONTENTS.

PAGE

**The Enemy of the Race** ... ... ... ... ... 473

**The Informed Verdict** ... ... ... ... 474

**Hospital and Institutional News** ... ... ... 475

The Retirement of Sir Alfred Keogh—The Torpedoing of the *Glenart Castle*—Hospital Expansion in West Ham—The Bradford War Memorial—British Hospitals Association—The Site of St. Mark's Hospital—Arundel Hospital: Past and Present—A Successful Cottage Hospital—A New Hospital for North China—News from Palestine—An Object-Lesson in Persistence—The Immense Value of Gifts in Kind—Death of a Hospital Secretary—Cheltenham Hospital's New Secretary—The Salaries of Medical Superintendents—Milk Extravagance in Officers' Hospitals—The Future of the Maimed Soldier—Discharged Soldiers as Attendants—Rations in Sanatoria—This Week's Drug Market.

**The Great War** ... ... ... ... ... ... 479

III. The Expeditionary Force Base.

**Liverpool a Pioneer in Nursing** ... ... ... 481

Miss Agnes Jones' Great Work.

**Paralysis Following Arterial Injuries** ... ... 482

Dangers from Ligature of Main Arteries.

**Lord Lister** ... ... ... ... ... ... ... 483

VII. Antisepis and Asepsis.

**Prince of Wales' Hospital, Cardiff** ... ... 485

Opening by the Prince in Person.

PAGE

**Parliament and the Profession** ... ... ... 484

**The After-Care of the Disabled Soldier** ... 486

By Colonel A. W. Mayo Robson, A.M.S., C.V.O., C.B., F.R.C.S.

**The London Hospital** ... ... ... ... ... 487

Work of the Past Quarter.

**King's College Hospital** ... ... ... ... 487

**Nursing Affairs in Ireland** ... ... ... ... 488

Democratic Ideals.

**The Matrons' and Sisters' Department** ... 489

Nursing Progress and its Developments: IX. The Local Centres of the College of Nursing.
Round the Hospitals.
The Torpedoed Nurses—The College Council Elections—How to Nominate Candidates—American Nurses' Aides—Paltry Annuities for Nurses—A Super-Matron with Sarah Gamp.
Food Problem Notes.

**The College of Nursing (Limited by Guarantee)** 493

Meeting at the Dreadnought Hospital.

**Matrons' and Sisters' Appointments** ... ... 494

**Gifts and Bequests** ... ... ... ... ... ... 494

**The Institutional Worker** ... ... (Suppl.) 1

Crockery Washing in a Nurses' Home: Answer to January Question.
How to Sterilise Rubber Gloves: Answer to February Question.

